# Role of intervention programs to increase influenza vaccination in Israel

**DOI:** 10.1186/2045-4015-3-13

**Published:** 2014-04-25

**Authors:** Dan Yamin, Arieh Gavious, Nadav Davidovitch, Joseph S Pliskin

**Affiliations:** 1 Department of Industrial Engineering and Management, Ben Gurion University of the Negev, Be’er Sheva, Israel; 2 School of Management, Ono Academic College, Kiryat Ono, Israel; 3 Department of Health Systems Management, Ben Gurion University of the Negev, Be’er Sheva, Israel

**Keywords:** Influenza vaccination, Vaccination intervention programs, Incentives, Vaccination behavior, Risk perception

## Abstract

**Background:**

Influenza vaccination is the most efficient and cost-effective method to prevent influenza. To increase vaccination coverage, health authorities use various intervention programs (IPs), such as cost subsidies or placing vaccination centers in malls to make vaccination more accessible. Nevertheless, vaccination coverage has been sub-optimal in most developed countries, including in Israel.

**Methods:**

To determine possible drivers of individual vaccination uptake and to examine the effectiveness of different IPs in increasing vaccination, we analyzed a telephone survey of a representative sample of the Israeli population conducted in March 2011 (n = 470), and paper questionnaires at the work place and at homes during April-July 2011 to several sub-populations : soldiers (n = 81), medical staff (n = 107), ultra-orthodox Jews (n = 72), Israeli Arabs (n = 87) and students (n = 85).

**Results:**

The population can be stratified into three sub-groups: *Acceptors*, who receive vaccination regardless of IPs (22%), *Conditional Acceptors*, who are only vaccinated because of IP implementation (44%) and *Non-Acceptors*, who are not vaccinated despite IP implementation (34%). Our analysis shows that the risk perception towards influenza relative to vaccination is higher in the *Acceptors* than in the *Conditional Acceptors*, with the *Non-Acceptors* showing the lowest risk perception (P < 0.01). For *Conditional Acceptors,* physician recommendation is the most effective IP, regardless of the sub-population tested (P = 0.04). Students and low-income participants were more prone than any others to be persuaded to receive vaccination following IPs. In addition, financial incentives were more effective for ultra-religious orthodox Jews and students; vaccinations in more accessible areas were more effective for the ultra-religious orthodox, soldiers, and medical personnel; and TV and radio advertisements were more effective for people above 50 relative to other age groups.

**Conclusions:**

Risk perception of influenza and vaccination governs the likelihood of successful implementation of IPs. Policy makers in Israel should invest efforts to increase the knowledge regarding influenza and vaccination, and should apply specific interventions customized to the preferences and diverse perceptions among the Israeli sub-populations.

## Background

Influenza has a long history of causing substantial morbidity, mortality, and economic losses [1-3]. In Israel, influenza is responsible each year for about 801,200 reported infections (around 10% of the population), 4130 hospitalizations, 1140 deaths, 2.7 million work days lost, and an overall cost to the Israeli economy of 261 million dollars (~0.1 of the GDP) [3,4].

Vaccination is the primary method of preventing influenza infection. Vaccination is beneficial not only for the vaccinated, but also for the entire population due to reduced transmission [2]. Thus, recommendations of both the US CDC and the Israeli Ministry of Health encourage all individuals older than six months to be vaccinated. In particular, as influenza is most prevalent in children under five, and complications occur predominately in the elderly and in individuals with co-morbidities [5], vaccination is highly encouraged among these sub-populations [6].

To increase vaccination rates, authorities and healthcare providers also apply different forms of intervention programs (IPs). For example, one form of an IP could be to waive the vaccination fee. Another form could be to improve accessibility by placing immunization centers in malls or near work places.

In Israel, since 2008, influenza vaccination is provided for free to all age groups, and vaccination in the form of a nasal spray is partly covered [7]. Further, both the Ministry of Health and health maintenance organizations invest various efforts to promote vaccination, in particular, among targeted high-risk populations through TV, radio advertisements and physician recommendations. Although vaccination rates are estimated to range only between 15-20% among the entire population, the overall vaccination coverage is gradually increasing and is substantially higher among the targeted high-risk age groups, with 23-32% in children between six months to four years, 26-31% in the elderly between 50–65, and 58-63% in individuals above 65 [8]. Nevertheless, influenza vaccination coverage remains sub-optimal in the majority of developed countries including Israel [6,9,10].

The decision whether or not to be vaccinated is personal and does not necessarily take into account the welfare of others [11-13]. Health behaviour models suggest that individuals make their vaccination decisions based on risk perceptions [14]. From an individual perspective, vaccination can be an unpleasant procedure, which demands time, and, in countries where vaccination is not funded, money. Several other factors such as religious and personal beliefs regarding vaccinations, perceived vulnerability, perceived susceptibility, vaccination efficacy, disease severity [14], effects of worries and regrets [15], vaccine criticism by media and, sometimes, even by health care workers, all have been shown to account for the relatively low vaccination rates [10,16].

Earlier studies suggested that the influenza vaccination decision correlates mainly with vaccination efficacy, disease likelihood, and disease severity relative to vaccination severity [14,17]. Other studies have focused on the question of which IPs are most effective at increasing vaccination rates [6,18]. However, to our knowledge, no study to date has integrated the relationship between the effectiveness of IPs on individual vaccination decisions with their risk perceptions about influenza and vaccination to examine the combined effect on vaccination rates.

To determine the main drivers for vaccination uptake and to examine the effectiveness of different IPs in increasing vaccination uptake, we evaluated perceptions of the Israeli population through a telephone survey of a representative sample of the Israeli population as well as through paper questionnaires distributed to sub-groups of interest. Based on the obtained perception towards influenza and vaccination, we found that the population can be divided into three sub-groups: *Acceptors* who were vaccinated regardless of IPs, *Conditional Acceptors* who were likely to be vaccinated only due to implementation of IPs, and *Non-Acceptors* who did not receive vaccination despite IPs. Specifically, we show that to increase compliance, policy makers should focus mainly on efforts to increase the knowledge about influenza and vaccination and apply IPs that are customized to the preferences of the sub-populations. By helping to improve current IP prioritizations in Israel, our results stand to inform optimal resource allocation for maximal influenza vaccination coverage, thus advancing overall public health.

## Methods

### Participants

The telephone survey was conducted by the B. I. Cohen Institute for Public Opinion Research in March 2011. The timing was chosen at the end of the influenza season to prevent time bias as much as possible [19]. The questionnaire required short answers and multiple choice questions whereby all questions were mixed to prevent order bias in the multiple-choice questions. The sample included 917 individuals above 18 years of age from a representative sample of Israeli households. In order to achieve a representative sample, we used stratified sampling based on socio-demographic characteristics that included the following: geographic regions, years in the country, levels of religiosity and socio-economic levels (salary and formal education). The stratifications were composed according to data taken from the Israeli Central Bureau of Statistics. The telephone interview was conducted either in Hebrew, Russian (to make the sample more representative especially among older immigrants from the former Soviet Union who are not Hebrew speakers), or Arabic. In cases in which the phone calls were unanswered, pollsters repeated calls up to five times within a period of three weeks. If calls were not successful by the fifth time, another individual from the same sub-population was chosen. However, cellular phone numbers were not included. Out of the 917 interviewees, 83 were unreachable after the procedure was initiated. Thus, our representative sample size contained 834 subjects. From that sample, 364 refused to participate or did not fully cooperate leading to 470 interviewees (response rate 56.7%). Among them, 192 were elderly above 50 years of age, and 244 were parents to children less than 18 years old.

The paper questionnaires for special interest groups were distributed in person during April-July 2011. The participants filled the questionnaires while the pollsters stood by them. The interest groups chosen included a convenience sample of 81 soldiers who were beyond their mandatory service (97.6% response rate) serving in five different bases located in central and southern Israel, 85 students (80.6%) from four different departments (not related to health studies) at Ben-Gurion University of the Negev, 107 healthcare workers (61.8%) from two hospitals and three medical centers, 87 Israeli Arabs (77.6%) from 8 different towns and 72 ultra-orthodox Jews (76.5%) from five cities and towns. Being convenience samples, they may not reflect a representative sample of each sub-population.

### Procedure

In all surveys, individuals were first asked questions to determine their perceived risks and hazards due to influenza and vaccination (see Additional file 1). To better capture individual perceptions of the matter, we phrased the questions using the words “according to your feelings”, as these were shown to be better predictors than those phrased as purely cognitive probability judgments [17].

Individuals were then asked if they intended to be vaccinated in the next influenza season, and the major reasons for their decisions. In addition, for each interviewee, we presented ten types of IPs (Table 1), and asked the interviewee to state for each IP, on a seven point Likert scale, his/her tendency to get vaccinated if the intervention program was to be applied, where *1* means ‘won’t persuade me to take the vaccination’, and *7* means ‘will definitely persuade me to take the vaccination’. The IPs were presented in a random order to prevent potential bias. In 2011 several IPs had already been implemented (see introduction). To take this effect into the individual’s vaccination decision we assumed in our analysis that an IP will be effective if one of two conditions was observed: 1) an individual declared he/she would not be vaccinated in the upcoming season, but indicated he/she will be willing to do so if the IP is offered; 2) an individual declared he/she will be vaccinated in the upcoming season because of the IP that is currently offered. We present here results obtained under the assumption that IPs are effective if an individual marked 6 or 7 on the Likert scale. The same trends were found when we assumed that IPs are effective only for a score of 7 or for a score of 5 and above on the Likert scale (available from the authors).

**Table 1:** Intervention programs offered in the survey study

**Number**	**Questionnaire item of the intervention program**
1.	You will receive the vaccination for free
2.	Vaccination will be provided as a nasal spray rather than a shot
3.	You will receive vaccination in a close and more convenient place, such as a mall or a location near the work place
4.	You will receive a $12* coupon to use as you wish in a pharmacy
5.	You will receive $12^*^ if you take the vaccination
6.	You will receive information pamphlets regarding the disease and regarding the vaccination
7.	Your family doctor will recommend vaccination
8.	TV and radio advertisement will encourage vaccination
9.	You will receive a phone call reminder to be vaccinated
10.	You will be assessed a health tax of $12 if you do not get vaccinated

^*^All prices are translated to U.S. Dollars but originally were presented in the local currency, New Israeli Shekels (NIS).

### Data analysis

To consider our hypothesis that risk perception towards influenza and vaccination affects the vaccination decision differently when an IP is provided or not, we used an ordinal regression model (using the Polytomous Universal procedure). Specifically, we divided the population into three ordinal categories based on their behavior: 1) *Acceptors*; 2) *Conditional Acceptors*, and; 3) *Non-Acceptors*. The model is represented as follows:

$$\begin{array}{cc} \ln\;\left(\theta_{j}\right)=\alpha_{j}-\beta_{1}X_{1}-\beta_{2}X_{2}-\beta_{3}X_{3} & j=1,2 \end{array}.$$

For each interviewee we evaluated risk perceptions as the three independent variables in the model: perceived risk reduction following vaccination, *X*_*1*_; perceived likelihood of infection if the individual is not vaccinated, *X*_*2*_*;* and perceived severity differences between infection and vaccination, *X*_*3*_ (see questionnaire items in Table 2). *β* represents the corresponding coefficient for each independent variable. The logit, denoted ln(*θ*_*j*_), represents the natural logarithm of the odds for an individual in category *j* or less to be vaccinated compared with individuals from category greater than *j. α*_*j*_ represents the threshold value for each logit.

**Table 2:** Perceived values for the three groups

**Perceived values**	Questionnaire items^*^	***Acceptors *** **(22%)**		***Conditional acceptors *** **(44%)**		***Non-acceptors *** **(34%)**	
		**Mean**	**95% CI**	**Mean**	**95% CI**	**Mean**	**95% CI**
**Perceived likelihood of infection**	Generally speaking, what are the chances that you will contract seasonal influenza if you do not get vaccinated in that season?	6.57	(6.24, 6.89)	5.22	(4.75,5.70)	4.16	(3.82,4.49)
Perceived risk reduction^**^	a. Generally speaking, what are the chances that you will contract seasonal influenza if you get vaccinated in that season?	3.13	(2.72,3.53)	1.61	(1.01,2.21)	0.45	(0.06, 0.83)
	b. Generally speaking, what are the chances that you will contract seasonal influenza if you do not get vaccinated in that season?						
Perceived severity^**^	a. Do you feel that it is dangerous for you to contract seasonal influenza?	3.71	(3.26,4.16)	1.03	(0.42,1.64)	0.06	(−0.38,0.50)
	b. Do you feel that it is dangerous for you to get influenza vaccination?						

*All questions were ranked on a 10 point Likert scale where 0 means no chance of becoming infected (no hazard at all) and 10 means definitely will become ill (extremely high hazard).

**The perceived risk and severity reductions were computed as the difference between the two questionnaire items. The difference could be negative as several interviewees pointed out that vaccination might be more harmful than infection.

To gain further insight into the relationship between risk perception and vaccination behavior, we conducted Tukey post-hoc tests for each of the three variables of risk perception, as the dependent variables, and vaccination decision (i.e., *Acceptors*, *Conditional-Acceptors*, and *Non-Acceptors*), as the factor. Further, the Pearson correlation was used to determine the correlations between the receptiveness of an individual to become vaccinated for the different IPs provided.

## Results

To determine vaccination uptake and examine the effectiveness of different IPs in increasing vaccination uptake, we stratified the population into three sub-groups: *Acceptors,* who receive vaccination regardless of IPs (22%), *Conditional Acceptors,* who will be vaccinated only due to implementation of IPs (44%) and *Non-Acceptors,* who are not vaccinated despite IPs (34%). Our analysis of the ordinal regression model shows that the risk perception towards influenza relative to the risk of the vaccine is significantly different among the three groups (P < 0.01).

Specifically, the risk perception towards influenza relative to vaccination is higher in the *Acceptors* than in the *Conditional Acceptors*, with the *Non-Acceptors* showing the lowest risk perception. This finding suggests that risk perceptions may play an important role in understanding which group is more likely to be persuaded by the implementation of intervention programs.

For each interviewee, we evaluated the perceived severity reductions by computed the difference between his/her perceived severity of infection and the perceived severity of vaccination (Table 2). The difference could be negative as several interviewees pointed out that vaccination might be more harmful than infection. Likewise, we evaluated the perceived risk reductions of infection following (Table 2). Post-hoc homogenous tests intensify these findings, and demonstrate that these three sub-groups are significantly different from each other in terms of individuals’ perceived likelihood to become infected with influenza (Tukey, P < 0.01), the perceived risk reduction following vaccination (Tukey, P < 0.023), and the perceived severity of infection compared to vaccination (Tukey, P < 0.01) (Table 2). Moreover, among the *Non-Acceptors*, 41.3% (95% CI, 36.8-45.7%) declared that vaccination is more harmful than being infected with influenza.

**Figure 1:**
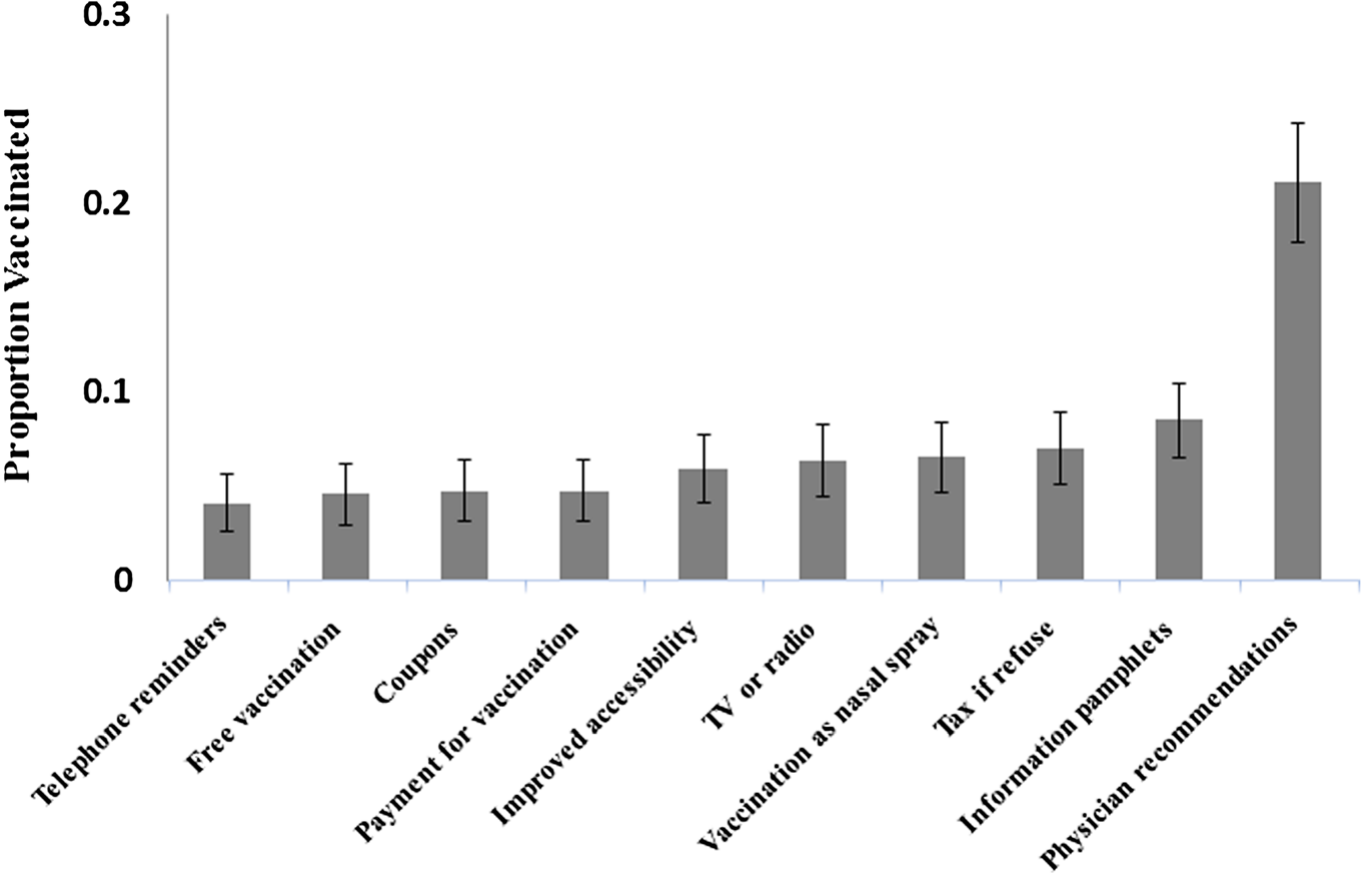
**Proportion of total population vaccinated given different intervention programs.** Mean proportion and 90% confidence level for the overall population given IPs.

For *Conditional Acceptors*, our results indicate that the form of the IP has a moderate effect on the vaccination decision. Namely, individuals who are more likely to be vaccinated due to the provision of a certain IP are more likely to be vaccinated if another IP would have been provided instead (0.22 < r^2^ < 0.62, P < 0.001). In the overall population, we also found that physician recommendations were the most effective IPs (Figure 1). The provision of information pamphlets regarding the disease and regarding the vaccination was found as the second most influential IP, yet not significant than the rest of the IPs (P = 0.29).

Our analysis of the special interest sub-populations we interviewed demonstrates that some sub-populations are more prone to accept vaccinations (Table 3). For example, 81% of the students who were not willing to be vaccinated without IPs (69) would be persuaded to be vaccinated if they received proper IPs. However, only 30% of healthcare workers would be persuaded to be vaccinated if proper IPs were provided. If no IPs were provided, 33.9% of the healthcare workers and only 8.1% of the students would be vaccinated. Additionally, in line with previous studies [20], vaccination coverage among Israeli Arabs was as high as 30% regardless of IPs (Table 3).

**Table 3:** Comparison among sub-populations

	**Paper questionnaires**					**Segments from the telephone survey**			
**Sub-population**	**Ultra-orthodox Jews N = 72**	**Soldiers N = 81**	**Students N = 85**	**Israeli Arabs N = 87**	**Health-care workers N = 107**	**Parents of children below 18 N = 244**	**Elderly above 50 N = 192**	**High**^**a **^**level income N = 161**	**Low**^**a **^**level income N = 195**
*Descriptive*									
Age (Years)	37.3 (9.5)	36.1 (6.1)	24 (1.8)	38 (14)	39 (11)	53.7 (20.1)	63.3 (10.1)	45 (16)	49.6 (18)
Gender (percentage female)	61.7 (5.1)	14.8 (3.9)	64 (5.1)	52 (5.3)	79 (3.8)	43.5 (3)	44.2 (3.5)	50.1 (4)	54.4 (3.6)
*Categories (percentage)*									
Self- Acceptors	20 (4.4)	11 (3.4)	8 (3)	31(5.1)	33 (4.5)	28 (3)	19 (2.6)	24.4(3)	28.7 (3.2)
Intervention Program vaccinators	47 (5.5)	49 (5.5)	81(4.3)	41(5.4)	30 (4.3)	40 (3.2)	44 (3.3)	31.1(3.6)	41.5 (3.5)
Non-Acceptors	33 (5.2)	4 (5.4)	11(3.4)	28(4.9)	37 (4.6)	32 (3.1)	37 (3.2)	44.5 (4.4)	29.8 (3.2)
*Form of intervention programs (percentage willing to be vaccinated)*									
Free vaccination	16.1 (4.1)	21 (4.5)	18.8 (4.2)	9.2 (3.1)	8.3 (2.7)	7.1 (1.7)	6.3 (1.74)	3 (1.3)	7.7 (2)
Improved accessibility	21 (4.5)	25.9 (4.8)	24.7 (4.6)	12.6 (3.6)	11 (3.1)	6.7 (1.6)	4.7(1.5)	5.6 (1.8)	8.2 (2)
Vaccination as nasal spray	13.6 (3.8)	23.5 (4.7)	22.4 (4.5)	11.5 (3.4)	11 (3.1)	6.7 (1.6)	5.7 (1.7)	7.4 (2)	7.1 (1.8)
Physician recommendations	28.4 (5)	28.4 (5)	25.9 (4.7)	28.7 (4.9)	18.3 (3.8)	28.4 (2.9)	31.3 (3.3)	20.4 (3)	29.7(3.1)
Coupons	14.8 (3.9)	19.8 (4.4)	23.5 (4.5)	10.3 (3.3)	6.4 (2.4)	6.2 (1.5)	4.7 (1.5)	2.4 (1.2)	8.7 (2)
Telephone reminders	14.8 (3.9)	18.5 (4.3)	8.2 (2.9)	6.9 (2.7)	7.3 (2.5)	5.3 (1.4)	3.1 (1.2)	3.1 (1.3)	6.6 (1.7)
Tax if refuse	13.6 (3.8)	13.6 (3.8)	54.1 (5.3)	13.8 (3.7)	10.1 (2.9)	9.3 (1.9)	6.3 (1.7)	5.5 (1.8)	9.8 (2.1)
TV or Radio	8.6 (3.1)	9.9 (3.3)	9.4 (3.1)	11.5 (3.4)	9.2 (2.8)	7.6 (1.7)	8.3 (2)	7.5 (2.1)	8.2(2)
Information pamphlets	18.8 (4.3)	18.5 (4.3)	11.8 (3.5)	14.9 (3.8)	10.1 (2.9)	10.7 (2)	6.8 (1.8)	8.7 (2.2)	8.7(2)
Payment for vaccination	18.5 (4.3)	21 (4.5)	42.4 (5.3)	12.6 (3.6)	6.4 (2.4)	6.2 (1.5)	6.3 (1.7)	3.1 (1.3)	9.2 (2.1)

^a^High and low incomes refer to a rank of an individual’s household salary compared to the gross median salary of households in Israel in 2012.

Financial incentives such as waiving the vaccination fee or providing coupons to vaccinated individuals were more effective than others for ultra-religious orthodox Jews and for students; vaccinations in more accessible areas were highly effective for the ultra-orthodox Jews, soldiers, and medical personnel; and TV and radio advertisements were highly effective for people above 50 years of age (Table 3). Individuals with lower income levels were more receptive (P = 0.025) to IPs than individuals with higher income levels. Those with lower income levels were also more affected by financial incentives (Table 3). Gender was not found to affect the results.

## Discussion

In line with previous studies, we showed that risk perceptions governed influenza vaccination decisions [14,21-23]. We further determined that risk perceptions can also help predict whether IPs would be effective in altering vaccination behavior. Our findings suggest that the Israeli population as a whole has a solid opinion on vaccination. Thus, rather than targeting the entire population, IPs may influence best the *conditional acceptors* who have an intermediate perception towards the risk. Thus, IPs should be designed to consider the IP’s preferences of this sub-population in mind.

In line with previous studies [24-26], our analysis suggests that physician recommendation is the most effective intervention program in nearly all sub-groups tested. However, this form of intervention might be more costly for implementation. On the other hand, the provision of information pamphlets regarding both the disease and the vaccination is fairly cheap and highly effective. Regardless of the form of intervention, our results suggest that the socio-demographic and socio-economic diversity in the Israeli population may necessitate that IPs be customized to the preferences of each sub-population. For example, TV advertisements should focus mainly on the elderly, whereas vaccination centers would be effective if provided in proximity to the habitants of Ultra-orthodox Jews. Importantly, evaluation of the effectiveness of a combination of IPs could not be accurately achieved through our survey. Future research could use case control studies with larger number of participants than conducted in this study to analyze the influence of combined IPs on vaccination rates among each sub-population.

Former studies have shown that people tend to try to satisfy the pollsters in survey studies [27], which for our study would imply that the effect of IPs may be less desirable than we observed. The overall declared vaccination coverage obtained through the telephone survey was 14% higher than the actual coverage [8]. Further, risk perceptions and attitudes regarding IPs may change over time and should be evaluated frequently. For example, low uptake and arguments for rejecting the A/H1N1 vaccine were observed in 2009 in Israel [22], whereas in 2013 a higher demand for the influenza vaccine was reported in the beginning of the season, possibly due to increased awareness following a successful polio vaccination campaign [28].

## Conclusion

In conclusion, we showed that risk perception of influenza and vaccination governs the likelihood of successful implementation of IPs. Our results indicate that policy makers in Israel should invest efforts to increase the knowledge regarding influenza and vaccination, and apply specific interventions customized to the preferences of the diverse Israeli sub-populations.

## Competing interests

The authors declare that they have no competing interests.

## Authors’ contributions

DY: data analysis, data collection, manuscript preparation, survey studies design. AG: manuscript preparation, data analysis. ND: survey studies design, manuscript preparation. JP: survey studies design, manuscript preparation. All authors read and approved the final manuscript.

## Supplementary Material

Additional file 1 Supporting information. Click here for file
