# Supplementary material for: Role of intervention programs to increase influenza vaccination in Israel
Source: Isr J Health Policy Res. 2014 Apr 25;3:13. doi: 10.1186/2045-4015-3-13 (PMC4021634; doi:10.1186/2045-4015-3-13)
Supplement: Additional file 1 — Supporting information. [file 2045-4015-3-13-S1.doc]

**Supporting information**

Presented here are the relevant questions from the telephone survey. [Note that the order of questions and multiple-choice answers, within and between groups was mixed to prevent bias]:

**Questions Group A**

Answer the following questions on a scale from 0 to 10, where 0 means "I will definitely not become infected" and 10 means "I definitely will become infected":

1. Generally speaking, what are the chances that you will contract seasonal influenza if you are not vaccinated in that season?

2. Generally speaking, what are the chances that you will contract seasonal influenza if you are vaccinated in the same season?

Answer the following questions on a scale from 0 to 10, where 0 means "no hazard at all" and 10 means "extremely high hazard":

3. Do you feel that it is hazardous for you to contract seasonal influenza?

4. Do you feel that it is hazardous for you to get the influenza vaccination?

**Questions Group B**

Please answer the following multiple-choice questions:

5. In the past six months, were you vaccinated for influenza? A. yes; B. no; C. don't remember or refuse to answer.

6. Before next fall, will you receive the influenza vaccination? A. yes; B. no; C. don't know or refuse to answer.

For those who answered 'A' in 6, what is/are the main reason/s for your answer? A. Family doctor recommendation; B. I am afraid of contracting the disease; C. Radio or TV advertisements; D. In my household, I live with children under 5; E. In my household, I live with elderly above 65; F. I have a weak immune system; G. Because of recently published mortality incidence; H. Vaccination is provided for free; I. Other ______

For those who answered B. or C. in 6, what is the main reason for your decision?

1. I am afraid of shots; B. Usually I am healthy; C. I do not believe in influenza vaccination; D. My family doctor did not recommend influenza vaccination; E. I do not consider it an important issue; F. No particular reason.

**Questions group C**

I will read you a few sentences. For each sentence please address the likelihood it will persuade you to be vaccinated where '1' means “will not persuade me to be vaccinated” and ‘7’ means “will definitely persuade me to be vaccinated”.

1. You will receive the vaccination for free
2. Vaccination will be provided as a nasal spray rather than a shot
3. You will receive the vaccination in a close and more convenient place, such as malls or near work places
4. You will receive a coupon valued at $12 to use as you wish in a pharmacy
5. You will receive $12 if you receive the vaccination
6. You will receive information pamphlets regarding the disease and regarding the vaccination

7. Your family doctor will recommend vaccination

8. TV and radio advertisements will encourage vaccination

9. You will receive a phone call reminder to receive the vaccine
